# Quantifying Effects of Pharmacological Blockers of Cardiac Autonomous Control Using Variability Parameters

**DOI:** 10.3389/fphys.2017.00010

**Published:** 2017-01-23

**Authors:** Renata Miyabara, Karsten Berg, Jan F. Kraemer, Ovidiu C. Baltatu, Niels Wessel, Luciana A. Campos

**Affiliations:** ^1^Center of Innovation, Technology and Education (CITE), Anhembi Morumbi University - Laureate International UniversitiesSao Jose dos Campos, Brazil; ^2^Center of Innovation, Technology and Education (CITE), Camilo Castelo Branco UniversitySao Jose dos Campos, Brazil; ^3^Institut für Physik, Humboldt-Universität zu BerlinBerlin, Germany

**Keywords:** autonomic nervous system, heart rate variability, symbolic dynamics

## Abstract

**Objective:** The aim of this study was to identify the most sensitive heart rate and blood pressure variability (HRV and BPV) parameters from a given set of well-known methods for the quantification of cardiovascular autonomic function after several autonomic blockades.

**Methods:** Cardiovascular sympathetic and parasympathetic functions were studied in freely moving rats following peripheral muscarinic (methylatropine), β1-adrenergic (metoprolol), muscarinic + β1-adrenergic, α1-adrenergic (prazosin), and ganglionic (hexamethonium) blockades. Time domain, frequency domain and symbolic dynamics measures for each of HRV and BPV were classified through paired Wilcoxon test for all autonomic drugs separately. In order to select those variables that have a high relevance to, and stable influence on our target measurements (HRV, BPV) we used Fisher's Method to combine the *p*-value of multiple tests.

**Results:** This analysis led to the following best set of cardiovascular variability parameters: The mean normal beat-to-beat-interval/value (HRV/BPV: meanNN), the coefficient of variation (cvNN = standard deviation over meanNN) and the root mean square differences of successive (RMSSD) of the time domain analysis. In frequency domain analysis the very-low-frequency (VLF) component was selected. From symbolic dynamics Shannon entropy of the word distribution (FWSHANNON) as well as POLVAR3, the non-linear parameter to detect intermittently decreased variability, showed the best ability to discriminate between the different autonomic blockades.

**Conclusion:** Throughout a complex comparative analysis of HRV and BPV measures altered by a set of autonomic drugs, we identified the most sensitive set of informative cardiovascular variability indexes able to pick up the modifications imposed by the autonomic challenges. These indexes may help to increase our understanding of cardiovascular sympathetic and parasympathetic functions in translational studies of experimental diseases.

## Introduction

Heart rate (HR) and blood pressure (BP) continuously fluctuate over time under the influence of control mechanisms such as autonomic nerve activity, humoral factors, and respiration for maintaining cardiovascular homeostasis. The investigation of heart rate and blood pressure variability (HRV and BPV) has become increasingly popular for the assessment of autonomic nervous system (ANS) such as autonomic tone, sympathovagal balance, or vagal function (Campos et al., 2013) in health and disease (Carthy, 2014). Various means of variability analysis have been employed such as time domain and frequency domain or linear and non-linear behaviors in order to find the HRV and BPV measures that could characterize best the ANS function in disease (Campos et al., 2013). For this, translational cardiovascular variability studies in controlled physiological conditions with pharmacological manipulation of the ANS function at different levels are necessary.

Time domain parameters are usually based on simple statistical methods; they are derived from the RR-intervals and their variations (Draghici and Taylor, 2016). The mean HR is the simplest parameter, but the respective standard deviation obtained from the whole-time series (sdNN) is the most prominent HRV measure for estimating overall HRV. The overall HRV estimate sdNN and other time domain parameters can be used to predict mortality in the recovery period after a myocardial infarction. A robust method to quantify the RR-distribution based on information theory is the Shannon entropy of the histogram.

Frequency domain HRV parameters allow the analysis of periodic dynamics in the HR time series (Draghici and Taylor, 2016). There are different techniques for spectral analysis: (1) methods based on Fast Fourier Transform (FFT) (Cerutti et al., 1991), (2) parametric autoregressive model estimation techniques (Rubini et al., 1993), and (3) wavelet approaches (Tsai et al., 2004; Neto et al., 2016). The results using different spectral methods, however, should be comparable (apart from differences in time and frequency resolution). The Task Force on HRV (Malik et al., 1996) recommends that power spectral analysis of 5-min ECG recordings should be used to assess autonomic physiology and pharmacology. The power within different frequency bands (see Table 1) can be estimated from such ECG recording. The high frequency power reflects modulation of vagal activity by respiration whereas the low frequency power represents vagal and sympathetic activity via the baroreflex loop. The low-to-high frequency ratio is used as an index of sympathovagal balance. Cross and slope 1/f denote the zero-crossing as well as the slope of the 1/f power-law scaling. For blood pressure (BP) analysis, all parameters described for HRV can be calculated for systolic blood pressure (SBP) and diastolic blood pressure (DBP) accordingly.

**Table 1:** **Description of time domain and frequency domain parameters**.

**Variable**	**Units**	**Definition**
**TIME DOMAIN STATISTICAL METHODS**		
meanNN	ms or mmHg	Mean BBI or mean blood pressure (BP)
sdNN	ms or mmHg	Standard deviation of all BBI or BP values
sdaNN1	ms or mmHg	standard deviation of 1-min averages
cvNN	None	sdNN/meanNN
RMSSD	ms or mmHg	Root mean square of successive BBI or BP differences
pNNlX (•)	%	Percentage of beat-to-beat differences lower than X ms (mmHg) [e.g., X = 3/6/9/12 ms (mmHg)]
shannon (•)	None	Shannon entropy of the histogram (density distribution of the BBIs or BP values)
**FREQUENCY DOMAIN METHODS**		
P	ms^2^ or mmHg^2^	Total power from 0−0.4 Hz
ULF	ms^2^ or mmHg^2^	Ultra low frequency band 0−0.05 Hz
VLF	ms^2^ or mmHg^2^	Very low frequency band 0.05−0.18 Hz
ULF+VLF	ms^2^ or mmHg^2^	Frequency band 0−0.18 Hz
LF	ms^2^ or mmHg^2^	Low frequency band 0.18-1 Hz
HF	ms^2^ or mmHg^2^	High frequency band 1-2 Hz
LF/HF	None	Quotient of LF and HF
HFn	None	Normalized high frequency band [HF/(LF+HF)]
LFn	None	Normalized low frequency band [LF/(LF+HF)]

*Adopted standards (Malik et al., 1996) and additional measures indicated by (•) are listed. BBI refers to the filtered beat-to-beat intervals (NN-intervals)*.

HRV and BPV reflect the complex interactions of many different control loops of the cardiovascular system. In relation to the complexity of the sinus node activity modulation system, a predominantly non-linear behavior has to be assumed. Thus, the detailed description and classification of dynamic changes using time and frequency measures is often not sufficient. Therefore, new methods of non-linear dynamics derived from the symbolic dynamics to distinguish between different states of the autonomic interactions were introduced (Voss et al., 1996; Wessel et al., 2007a). We and others have shown that tools from non-linear dynamics may improve the diagnosis in clinical conditions, such as a risk stratification for sudden cardiac death, forecasting of life-threatening cardiac arrhythmias, atrial fibrillation after cardiac surgery, congestive heart failure (reviewed in Wessel et al., 2007a).

Here, we aimed at investigating the cardiovascular variability indices relative to the sympathetic and parasympathetic components of ANS and identifying the most sensitive of them. Classical and newer variability analyses in time and frequency domains were applied to heart rate and blood pressure variability responses evoked by pharmacological manipulation of ANS.

## Methods

All procedures complied with guidelines from the American Physiological Society, and the institutional ethical committee approved the study (Camilo Castelo Branco University Ethical Committee approval no. 0021/2013).

Male Sprague-Dawley (SD) rats (12–14 weeks of age) were used for the study. Following acclimatization and a health examination, rats were housed in groups of three in standard cages with wire mesh tops and standardized softwood bedding, synchronized to a 12-h light–dark cycle, at ambient temperature 23 ± 2°C. A standard rat diet and tap water were supplied *ad libitum*. The rats underwent implantation of polyethylene catheters (PE-50; filled with 10 IU/ml heparinized saline) that were inserted into the femoral artery (to monitor arterial pressure and heart rate) and femoral vein (for i.v. injection of drugs) and exteriorized in the interscapular area and housed individually, as previously described (Baltatu et al., 2001). At least 48 h were allowed between the catheter implant and the experiment. Six rats per group for each drug were used.

All experimental protocols were performed between 2 and 4 pm (the period with lowest activity) in conscious, unrestrained and unstressed animals housed in individual cages. The experimental phase of the study investigated the effects of the following autonomic blockers at doses previously established to cause a BP or HR effect: Peripheral muscarinic (methylatropine, 0.5 mg/kg) (Woods et al., 1994), β1-adrenergic (metoprolol, 1 mg/kg) (Webb et al., 1991; Pham et al., 1993), α1-adrenergic (prazosin, 1 mg/kg) (Kurnjek et al., 1983; Pham et al., 1993), and ganglionic (hexamethonium, 10 mg/kg) (Gao et al., 1997) blockades. The effectiveness of the dosing (in 0.1 mL volume of injection) was shown through a sustained effect on heart rate or blood pressure. The measurements of BP and HR were performed with the catheter connected to a standard blood pressure transducer that was coupled to a PowerLab and recorded with LabChart 8 software (ADInstruments, Sydney, Australia). Intrafemoral blood pressure was sampled at 4 kHz to generate evenly spaced signals to monitor the arterial pressure and heart rate. Online monitoring was performed using Blood Pressure Module for LabChart software that automatically detects, analyzes and reports cardiovascular parameters from arterial pressure signals, including systolic/diastolic BP and beat-to-beat intervals (BBI, or NN-intervals detected automatically from maximal values of the BP oscillations). After recording a stable baseline period (30 min of stable hemodynamics), the drug was injected and further beat-to-beat measurements of BP and HR were performed for 60 min when the drug effect reached its peak (peak-time) in a plateau with stabilized hemodynamics.

### Cardiovascular variability analyses

The calculation of all variability parameters was performed on time series derived from the original blood pressure signal as described in Wessel et al. (2007b). Missing beats and erroneous detections were eliminated using the adaptive filter described in Wessel et al. (2007a). Standard methods of HRV and BPV analysis including time domain as well as frequency domain parameters were obtained; most of them are based on linear methods. A list of these parameters is given in Table 1. The selection of these parameters was based on the idea that one should always use the simplest and best understood methods first.

The first step of non-linear dynamics derived from the symbolic dynamics is the transformation of the time series into symbol sequences using symbols from a given alphabet. Certainly, some of the detail information is lost in this thresholding process, but the coarse dynamic behavior is retained. Comparing different kinds of symbol transformations, we found that the use of four symbols, as explained in Equation (1), was appropriate for our purpose. The time series *x*_1_*, x*_2_*, x*_3_*,…, x*_*N*_ is transformed into the symbol sequence *s*_1_*, s*_2_*, s*_3_*,…, s*_*N*_*, s*_*i*_ ∈ *A* on the basis of the alphabet A = [0,1,2,3]. The transformation into symbols by Equation (7) refers to 4 given amplitude ranges where μ denotes the mean beat-to-beat interval and *a* is a specific parameter which is heuristically determined.

(1) $$s_{i}(x_{i})=\{\begin{array}{cccc} 0: & \mu & <x_{i}\leq & (1+a)\mu \\ 1: & (1+a)\mu & <x_{i}< & \infty \\ 2: & (1-a)\mu & <x_{i}\leq & \mu \\ 3: & 0 & <x_{i}\leq & (1-a)\mu \end{array}\text{with }i=1,2,3,\ldots$$

Note, that the order of the symbols in formula (1) is of no relevance. There are several quantities that characterize such symbol strings. Analyzing words of length 3 (i.e., substrings which consist of three adjacent symbols) leads to a maximum of 4^3^ = 64 different words (bins). This word length is a compromise between retaining important dynamical information and having robust statistics, when estimating the probability distribution of the words. The Shannon entropy *H*_*k*_ calculated from the distribution *p* of words is the classic measure “FWShannon” for the complexity in time series:

$$\begin{array}{l} H_{k}=-\underset{\omega \in W^{k},p\left(\omega \right)>0}{\sum }p\left(\omega \right)\log p\left(\omega \right)\; \end{array}$$

(Malik et al., 1996)

where *W*^*k*^ is the set of all words of length *k*. Larger values of Shannon entropy refer to higher complexity in the corresponding tachograms and lower values to lower ones. “Forbidden words” (FORBWORD) in the distribution of words of length 3 are those words that never (or almost never) occur. A high number of forbidden words reflects a rather regular behavior in the time series. If the time series is highly complex in the Shannonian sense, only a few forbidden words are found. WPSUM02 (WPSUM13) denotes the percentage of words consisting only of the symbols “0” or “2” (“1” or “3”). WSDVAR is the overall word variability (Voss et al., 1996).

The parameter ‘POLVAR3’ characterizing short phases of low variability from successive symbols is determined by using another simplified alphabet *B* which consists of symbols “0” and “1,” only. Here “0” stands for a small difference between two successive values, whereas “1” represents cases when the difference between two successive data points exceeds a certain limit δ, specifically

(2) $$s_{n}=\{\begin{array}{cc} 1: & \left|x_{n}-x_{n-1}\right|\geq \delta \\ 0: & \left|x_{n}-x_{n-1}\right|<\delta . \end{array}\text{with }n=1,2,3,\ldots$$

Words consisting of a unique type of symbols (either all “0” or all “1”) are counted. To obtain a statistically robust estimate of the word distribution, words of length six, defining a maximum of 64 different words were chosen. “POLVAR3” represents the probability of the word “000000” occurrence and thus detects even intermittently decreased variability.

### Statistical analysis

Statistics were performed using R. Our initial test was a Wilcoxon paired test with a null hypothesis that there is no change between baseline and peak-time for the combination of measurement and calculated parameter. In order to select those variables that have a high relevance to, and stable influence on our target measurements (HRV, SBP, DBP) we used FisherÕs Method to combine the *p*-value of multiple tests. According to Fisher, the sum of the logarithms of the *p*-value times 2 is again χ^2^ distributed with 2^*^‘number of *p*-values’ degrees of freedom, so that we can again calculate a combined *p*-value.

## Results

**Figure 1:**
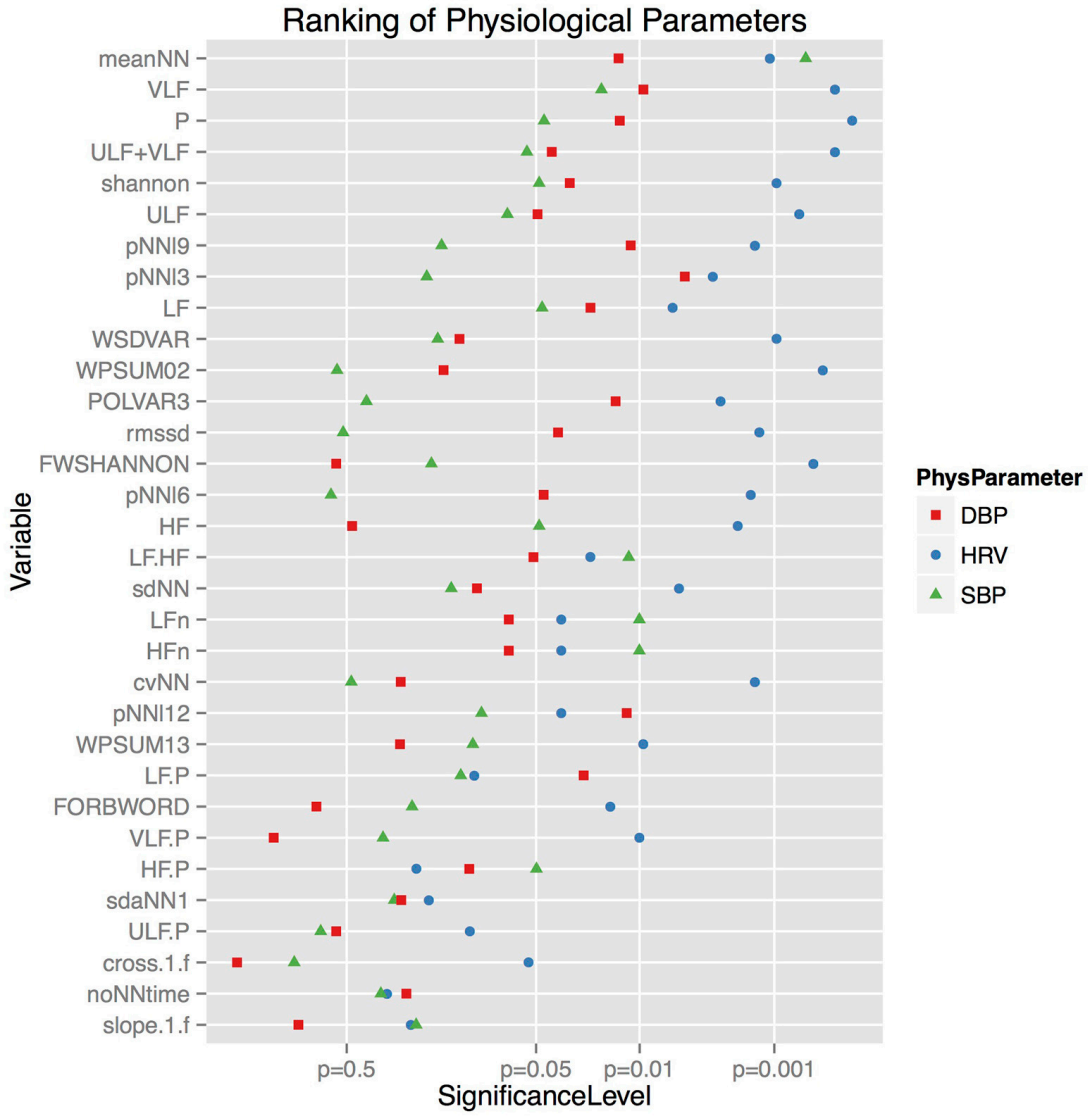
**The ability of different HRV, SBP, and DBP parameters to discriminate between the different autonomic blockades**. The significance levels shown below are achieved by the statistical analysis described in the respective section. The lower the *p*-value, the higher are the differences of the physiological parameter between the several blockades.

To show significant influences of the individual drugs on the cardiovascular variables we used standard analytical methods of time and frequency analysis (Malik et al., 1996) as well as from symbolic dynamics. Performing the statistical analysis described above we obtained 6 parameters that showed the biggest impact over all treatments between basal and peak time (cf. Figure 1).

The best set of measures were:

- FWSHANNON, the Shannon entropy of the word distribution,
- meanNN, the mean beat-to-beat interval or mean blood pressure,
- VLF, the very low frequency component of frequency domain analysis,
- POLVAR3, symbolic dynamics parameter to detect decreased variability,
- cvNN, the ratio of sdNN and meanNN and
- RMSSD, the root mean square differences of successive values.

As one can see from Figure 1, the biggest differences were found in HRV, more than half of the parameters were below the significance level of 0.01. The HRV complexity quantified by FWSHANNON showed the biggest differences between all blockades, however, the mean HR and BP values were highly significant, too. The VLF frequency band was significant for HRV and BPV. POLVAR3 was significant for HRV and DBP only, corresponding to a decreased HRV (<3 ms) and to a decreased diastolic BP variability (<3 mmHg). cvNN and RMSSD showed significant differences for HRV only.

Methylatropine affected mean HR and HRV, with an increase of POLVAR3 whereas all other parameters decreased due to decreased HRV (Table 2). Methylatropine had no effect on BP. For metoprolol, while the mean HR was not significantly altered, the HRV complexity and VLF component decreased. Metoprolol did not affect BPV although there was a decrease in mean BP. The combination of methylatropine with metoprolol led to a decreased mean HR. Moreover, the HRV complexity, VLF and POLVAR3 strongly decreased.

**Table 2:** **Set of the six best parameters showing the biggest impact over all treatments between basal and peak time**.

**Medication (HRV/BP)**	**FWSHANNON**	**VLF**	**POLVAR3**	**cvNN**	**meanNN**	**RMSSD**
Methylatropine	−−/0	−−/0	++/0	−/0	−/0	−−/0
Metoprolol	−/0	−−/0	0/0	0/0	++/0	0/0
Methylatropine + Metropolol	−−/0	−−/0	++/0	−/0	+/0	0/0
Prazosin	−−/0	−−/−−	++/++(D)	−/0	++/−−	−−/0
Hexamethonium	−/0	−−/−	+/0	−/0	0/−−	−/0

*Symbols: Big increase (++), increase (+), no change (0), decrease (−), big decrease (–). Changes for only one value of the diastolic (D) blood pressure are labeled (Prazosin/polvar3)*.

Prazosin was the only drug affecting significantly both HR and BP: Mean HR decreased, BP dropped, HRV complexity decreased, the VLF component fell down for both HRV and BPV and we saw a strong decrease in HRV and DBP (increase in POLVAR3).

Hexamethonium led to a drop in BP not affecting the mean HR, to a decreased VLF component in HRV and BPV and to a decrease in HRV (POLVAR3).

**Figure 2:**
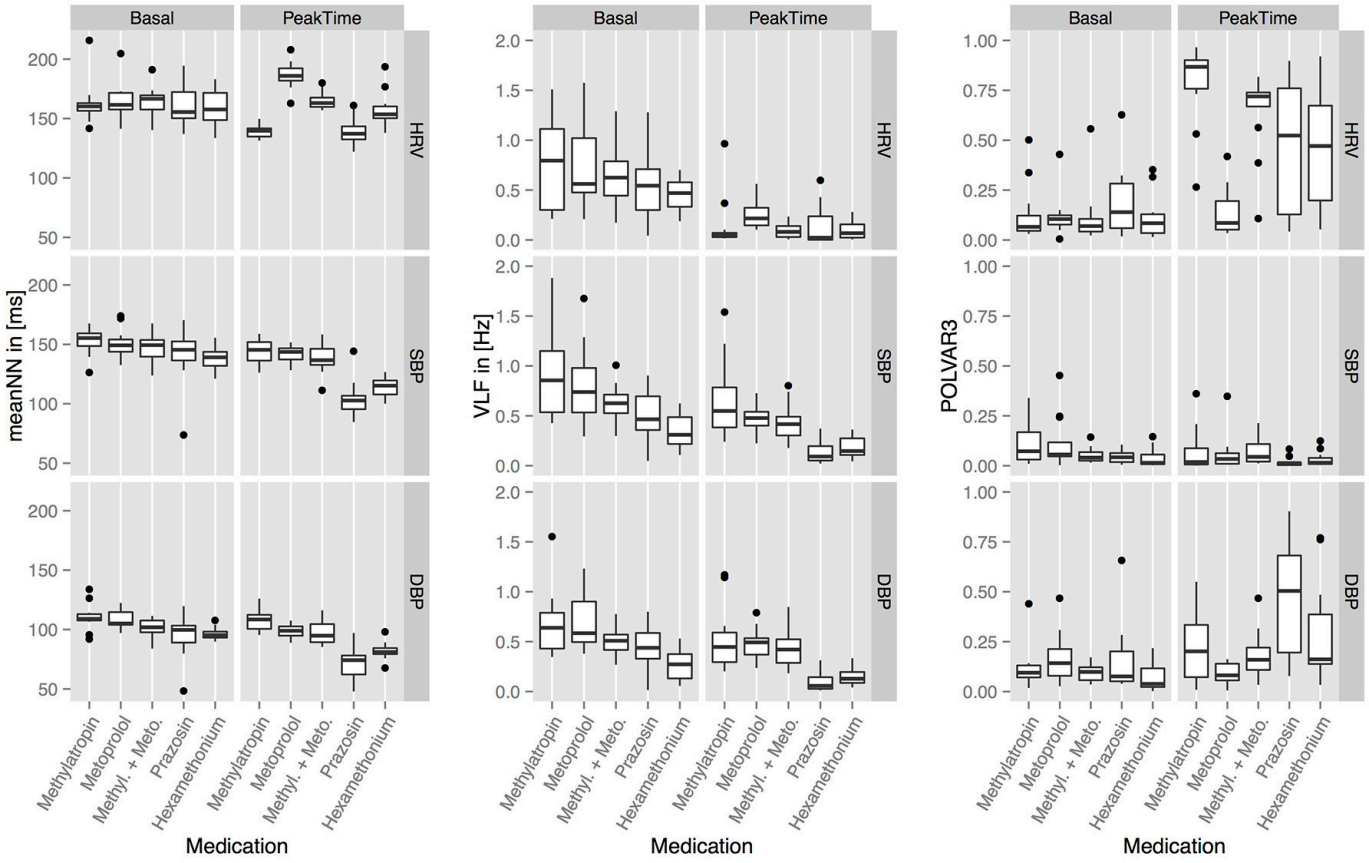
**The basal and peak time values for meanNN, VLF, and POLVAR3 for all five blockades and all domains (HRV, SBP, DBP)**.

In Figure 2 the basal and peak time values for the top 3 parameters: MeanNN, VLF, and POLVAR3 for all six blockades and all domains HRV, SBP, and DBP are shown. MeanNN represents the average changes in HR and BP, VLF, and POLVAR3 quantify intermittently decreased variability.

## Discussion

The present study provides the most sensitive measures of ANS function in rats that may best serve as biomarkers of ANS alterations in further translational studies. Throughout a complex comparative analysis of HRV and BPV measures altered by a set of autonomic drugs, we identified the most sensitive set of informative cardiovascular variability indexes able to pick up the modifications imposed by the autonomic challenges. These indexes may help to increase our understanding of cardiovascular sympathetic and parasympathetic functions in translational studies of experimental diseases. The Shannon entropy of the word distribution FWSHANNON and the symbolic dynamics parameter POLVAR3 appear as very sensitive measures to detect alterations in HR or BP variability induced by the ANS. Also, we do not expect that single measure can serve as a reliable biomarker for different complex diseases with overlapping (e.g., cardiovascular) symptomatology and this should be addressed in further studies. Instead, we aimed at identifying the most sensitive analytical method that could be used to detect the blockade of the specific arm of the ANS.

Symbolic dynamics has been applied to assess the autonomic cardiovascular regulation in men (Voss et al., 1996; Guzzetti et al., 2005) and experimental animals (Tobaldini et al., 2009a). Symbolic dynamics appears as more adequate than spectral analysis to analyze dynamic complexity of HRV in interpreting the complexity of short-term heart-period sequences in rats (Tobaldini et al., 2009b). It has been demonstrated in rats that symbolic analysis is a more sensitive tool than spectral analysis to track the changes in cardiovascular autonomic modulation (Tobaldini et al., 2009a). Symbolic analysis of SBP variability is consistent with those derived from HRV (Tobaldini et al., 2009a). There are further studies using symbolic analyses in animal models (Wernicke et al., 2007; Wessel et al., 2007b). A complete overview of all currently available symbolic parameters and a quantification of their interrelationship and dependence on certain medications were outside the scope of this paper. Our study indicates that symbolic dynamics derived parameter POLVAR3 and Shannon entropy of the word distribution FWSHANNON are sensitive measures of HR and BP variability to detect alterations in autonomic cardiovascular regulation in rats. In men, these profiles may be different due to the regular, periodic action of respiratory rhythm and to a more complex cardiovascular control at long time scales (Ho et al., 2011; Silva et al., 2016). Further studies shall certify whether HRV data in rats are equivalent homologous surrogates of cardiovascular morbidity and mortality (Farraj et al., 2011).

Alterations of the ANS tone during cardiac parasympathetic blockade were associated with a significant increase of the average HR. The ANS control of the heart rate has normally the parasympathetic (cholinergic) component dominant, meaning that the heart is under a tonic vagal activity. Therefore, the vagolytic activity of anticholinergic drugs causes an increase in heart rate and is used against sinus bradycardia and AV nodal block due to excessive vagal activation. Administration of atropine is the common method to investigate tonic vagal activity. Spectral analysis of HRV has shown that anticholinergic drugs generally decrease the HF power of HR and blood pressure variability in humans (Pomeranz et al., 1985; Parlow et al., 1997). While low doses of atropine cause a decrease in HR and an increase in HRV, higher doses cause an increase in HR and a decrease in HRV (reviewed in Lewis et al., 2006). These antagonizing cardiac effects of atropine occur since it can cross the blood-brain barrier and induce a central anticholinergic syndrome (Rupreht et al., 1983). In our study, we used methylatropine that is an exclusive peripheral antagonist that blocks the cardiac muscarinic receptors (M2) found mainly in sinoatrial node (Smith et al., 1994). Pharmacodynamics' data of methylatropine denote its higher vagolytic potency with a longer effect than atropine (Stoll, 1948; Jansen and Dellinger, 1989). In our study, methylatropine significantly affected the mean HR (increase) and HRV, without affecting BP. Of these HRV parameters, the best to characterize an inhibition of vagal tone was FWSHANNON, VLF, and RMSSD decrease, associated with a POLVAR3 increase.

The β-adrenoceptor antagonists (or β-blockers) are a class of sympatholytic drugs used for treating hypertension, angina, myocardial infarction, arrhythmias and heart failure (Frishman, 2008; Poirier and Tobe, 2014). Most of the studies on the cardiac autonomic effects of β-adrenoceptor antagonism in these cardiovascular diseases aimed at augmenting parasympathetic input through a reduction in sympathetic activity. These ANS β-blocker effects are largely acknowledged to be “cardioprotective” by decreasing the force and rate of myocardial contraction and renin secretion. To investigate the effects of sympathetic inhibition on HRV in our study we utilized metoprolol that is a β1-selective (cardioselective) adrenergic receptor blocker. Metoprolol acting on β1- adrenoceptors found in sinoatrial node slows the heart rate. In our study, the β-adrenergic blockade significantly decreased HR, and the VLF component of HRV.

The α1-adrenergic antagonists are exercising antihypertensive action by directly relaxing arterial and venous smooth muscle, and are used as adjuvant therapy in hypertension. Recent evidence is suggesting a cardioprotective role of α1-adrenergic receptors in the heart function in congestive heart failure (in the ALLHAT clinical trial, the arm of the trial using α1-blocker doxazosin had to be stopped because of higher number of heart failure events) (Cotecchia et al., 2015). Thus, the role and mechanisms of the “augmented of α-adrenergic tone in heart failure” (Leier et al., 1990) needs further investigation. In our study, prazosin was the only autonomic drug affecting both HR and BP levels. Acute blockade of the α-adrenergic tone with prazosin decreased blood pressure and increased heart rate. This was accompanied by a big drop of HRV and BPV complexity, as depicted by the increase of POLVAR3.

Ganglionic blockers were used in 1940s for “autonomic nervous system denervation” in resistant hypertension but they have been replaced due to their postural hypotension effect (DeQuattro and Li, 2002; D'Elia and Weinrauch, 2013). Of those, hexamethonium is a nicotinic cholinergic antagonist that does not cross the blood-brain barrier and is referred as prototype ganglionic blocker. Symptoms resembling ganglionic blockade occur in the autoimmune autonomic ganglionopathy (AAG), a rare disease (Garland et al., 2013). In our study, hexamethonium lowered mean BP without affecting mean HR. This was accompanied with a drop of VLF and HRV complexity and an increase of POLVAR3.

Some limitations of the present study must be addressed. While the HRV was calculated from the blood pressure curve and not from ECG, we showed that there is only a limited difference between the two methods of data acquisition (Suhrbier et al., 2006). In this study, the VLF spectral power from heart rate variability signal has been shown to be a sensitive marker of the autonomic nervous system activity (overcoming LF and HF spectral powers). However, while the origin and the significance of these oscillations are currently not completely understood, the results of our study can inform and should motivate further studies. Among the studied variability parameters, we observed that there was a decrease in time of the mean values and inter-individual variability of VLF SBP that could be explained through a recovery in time from the catheter implantation surgery or an adaptation of the animal to the experimental protocol. Also, this trend does not appear in any other parameters investigated. Besides, since the drug administration was in a given order, it could be thought that it might be a residual effect from the previous drug administration. However, the drugs have short half-lives (longest is 3 h) and 24 h between dosing allows a wash-out of the previous drug administration. Nevertheless, this should not affect the outcome of this study, as only the difference between basal and peak time was relevant.

In summary, we detected the most sensitive variability analysis derived measures of HR and BP after pharmacological modification of autonomic nervous system activity in rats. The translation of the findings from rats to human is not straightforward. The different basal setting of the ANS in rats should be taken into consideration in this process. As such species-specific differences include for instance the cardiovagal activity that is highest during inspiration in rats, while in humans it is inhibited (Draghici and Taylor, 2016). Further translational studies to assess ANS using these cardiovascular variability biomarkers in clinical practice are envisaged.

## Author contributions

Conceived and designed the experiments: OB, LC. Performed the experiments: RM, OB. Analyzed the data: KB, NW, RM, and JK. Wrote paper: OB, LC, NW, and RM. Edited manuscript: KB, JK.

### Conflict of interest statement

The authors declare that the research was conducted in the absence of any commercial or financial relationships that could be construed as a potential conflict of interest.
